# Capturing agro-morphological variability for tolerance to terminal heat and combined heat–drought stress in landraces and elite cultivar collection of wheat

**DOI:** 10.3389/fpls.2023.1136455

**Published:** 2023-05-09

**Authors:** Shubham Kumar, Hitesh Kumar, Vikas Gupta, Adesh Kumar, Chandra Mohan Singh, Mukul Kumar, Ajay Kumar Singh, Gurusharan Singh Panwar, Sujit Kumar, Akhilesh Kumar Singh, Rahul Kumar

**Affiliations:** ^1^Department of Genetics and Plant Breeding, Banda University of Agriculture and Technology, Banda, India; ^2^Crop Improvement Division, Indian Council of Agricultural Research (ICAR)-Indian Institute of Wheat and Barley Research, Karnal, India; ^3^Krishi Vigyan Kendra, Jhansi, Banda University of Agriculture and Technology, Banda, India; ^4^Uttar Pradesh (UP) Council of Agricultural Research, Lucknow, India; ^5^Department of Plant Science and Landscape Architecture, University of Connecticut, Storrs, CT, United States

**Keywords:** drought stress, elite cultivars, landraces, terminal heat stress, wheat

## Abstract

Climate change has resulted in extreme temperature and drought around the globe, which poses serious threat to food security. Both heat and drought stress affects the production and productivity of wheat crop. The present study was undertaken to evaluate 34 landraces and elite cultivars of *Triticum* spp. for phenological and yield-related traits under optimum, heat, and combined heat–drought stress environments during 2020–2021 and 2021–2022. The pooled analysis of variance showed significant genotype × environment interaction, suggesting an influence of stress on trait expression. The trait performance of genotypes exhibited significant reduction under combined heat–drought stress as compared to optimum and heat stress environments. The maximum seed yield penalty was observed under combined heat–drought stress environment as compared to heat stress alone. Regression analysis indicated significant contribution of number of grains per spike towards stress tolerance. Based on Stress Tolerance Index (STI), genotypes Local-17, PDW 274, HI-8802, and HI-8713 were identified to be tolerant to both heat and combined heat and drought stress at Banda, whereas genotypes DBW 187, HI-8777, Raj 4120, and PDW 274 were tolerant at Jhansi location. The genotype PDW 274 showed stress tolerance under all treatments at both the locations. The genotypes PDW 233 and PDW 291 showed highest stress susceptibility index (SSI) across the environments. The number of grains per spike and test kernel weight were positively associated with seed yield across the environments and locations. The selected genotypes Local-17, HI 8802, and PDW 274 were identified to be the potential sources of heat and combined heat–drought tolerance, which may be utilized in hybridization to develop tolerant wheat genotypes and also for mapping of underlying genes**/**quantitative trait loci (QTLs).

## Introduction

Wheat is the second most important cereal crop after rice, cultivated on approximately 30 million hectares with a production of 108 million tons in India (Annonymous, 2020). Globally, it is a vital staple food crop, feeding 2.5 billion people in more than 85 countries and a source of 20% calories in the human diet (Yashavanthakumar et al., 2021). Wheat productivity was reported to be affected up to 21% due to drought on a global scale from 1980 to 2015 (Daryanto et al., 2016). Global food security is threatened by the ever-increasing world population and by climate change (Lesk et al., 2016). The reduced rainfall, changing rainfall pattern, and distribution coupled with shorter winter seasons result in extreme temperature due to climate change (Lobell et al., 2011). Among abiotic stresses, drought and heat stress are the most important devastating factors for the growth and development of crop plants, affecting yield potential and end-use quality of food products (Delahunty et al., 2015). Furthermore, unpredictable yield losses by temperature and drought stress depends on annual precipitation patterns, surface water evapo-transpiration, and soil water holding capacity. The response of plants to water stress depends on several factors, such as developmental stage, intensity and duration of stress, and cultivar genetics (Wahid et al., 2007; Eskandari and Kazemi, 2010). Under water-deficit conditions, various physiological activities, particularly water and nutrient uptake, photosynthesis, partitioning of photosynthates, and metabolic activities, are affected, which ultimately results in significant yield loss (Farooq et al., 2009; Praba et al., 2009). Heat stress also impacts various metabolic activities like protein synthesis, inactivation of enzymatic activities, and physiological activities of the cell including cell membrane damage. The cell division is also adversely affected by heat stress (Smertenko et al., 1997). The damages from heat shocks seriously limit plant growth and favor oxidative damage. The terminal heat stress accelerates the assimilation of photosynthates due to early senescence, which results in shrunken grains and poor quality (Porter, 2005). Although the increasing temperature is also beneficial for crop production in some cooler regions of the world, the overall impact on global food security is still negative (Challinor et al., 2014).

Climate change has a negative impact on wheat production through extreme drought and temperature stress conditions during crop cycle. Hence, the identification of heat- and drought-tolerant genotypes is of paramount importance to the breeders. Phenotyping remains a key criterion for screening breeding materials based on drought-adaptive and constitutive morpho-physiological characteristics including yield and its components (Monneveux et al., 2012; Passioura, 2012). Seed yield and component traits-based physiological indices are widely used in wheat breeding programs for the selection of tolerant genotypes. Attempts to measure the degree of tolerance with a single parameter provide results of a limited value because of the multiplicity of the factors and their interactions contributing to drought tolerance under field conditions. Thus, there is a need to select genotypes with a good combination of agronomical and physiological important traits, cumulatively contributing to improved yields under target drought and heat environments (Tardieu, 2012). In combination, heat and drought stress harm plant growth and productivity more than any other environmental stress, although heat and drought stress individually have been extensively studied in wheat crop. A few studies were conducted to understand the plant response to high temperature during grain filling stage and in combination with drought stress conditions. The experimental sites in the Bundelkhand region are characterized by high temperature and water deficit during rabi season (rabi season is when the crops are sown around mid-November, preferably after the monsoon rains are over, and harvesting begins in April/May), and the studies on evaluation of wheat germplasm for heat and drought tolerance in Bundelkhand region are very limited. Generally, the crop is exposed to heat stress by planting late, and comparison is made with the normal planting, whereas the drought stress tolerance is estimated by comparing the performance of wheat genotypes under normal and water-deficit conditions (Liu et al., 2015; Gupta et al., 2020; Shokat et al., 2020). In the present study, the heat and combination of heat–drought stress tolerance was assessed to capture the agro-morphological variability for tolerance that could be utilized for developing tolerant wheat genotypes suiting the stress environments.

## Materials and methods

### Plant material and experimental site

The present investigation was carried out with 34 diverse wheat genotypes (**Table 1**), of which 18 were landraces collected across the Bundelkhand region (Uttar Pradesh); seven commercial durum cultivars were obtained from Indian Institute of Agricultural Research (IARI), Regional Research Station, Indore (MP), which are recommended for cultivation in Central Zone (CZ); seven durum cultivars were obtained from Punjab Agricultural University (PAU), Ludhiana (Punjab) representing Northwest Plane Zone (NWPZ), and two bread wheat cultivars were from IIWBR Karnal (Haryana). The landraces were collected from six districts (Banda, Mahoba, Chitrakoot, Jhansi, Hamirpur, and Lalitpur) of the Bundelkhand region, conserved by farmers for traditional cultivation. The local wheat collections possessed good adaptability to rainfed farming and are preferred by farmers of this region. The experiments were conducted for two consecutive crop seasons 2020–2021 and 2021–2022 at two locations (**Table 2**):

**Table 1:** Genotype name, cultivated spp., and source for collection for 34 wheat genotypes.

S. No	Genotype name	Cultivated spp.	Collection source
1	Local-1	*Triticum turgidum* ssp. *durum*	Sh. Shyam Babu,Village,Luktara, Banda
2	Local-2	*Triticum turgidum* ssp. *durum*	Sh. Arvind Pandey,Village,Luktara, Banda
3	Local-3	*Triticum turgidum* ssp. *durum*	Desi KhatiyaKVK, Hamirpiur
4	Local-4	*Triticum turgidum* ssp. *durum*	Desi KhatiyaKVK, Hamirpiur
5	Local-5	*Triticum turgidum* ssp. *durum*	Desi Khatiya, KVK Jalaun
6	Local-6	*Triticum turgidum* ssp. *durum*	Jalaun Local, KVK Jalaun
7	Local-7	*Triticum turgidum* ssp. *durum*	Desi Khatiya, KVK Jalaun
8	Local-8	*Triticum turgidum* ssp. *durum*	Desi Khatiya, KVK Lalitpur
9	Local-9	*Triticum turgidum* ssp. *durum*	Desi Khatiya, KVK Mahoba
10	Local-10	*Triticum turgidum* ssp. *durum*	Kathiya Surali, KVK Banda
11	Local-11	*Triticum aestivum* L.	TISI 306 Suroli,KVK Banda
12	HI-8759	*Triticum turgidum* ssp. *durum*	IARI, Regional Station, Indore
13	HI-8737	*Triticum turgidum* ssp. *durum*	IARI, Regional Station,Indore
14	HI-8713	*Triticum turgidum* ssp. *durum*	IARI, Regional Station, Indore
15	HI-8777	*Triticum turgidum* ssp. *durum*	IARI, Regional Station, Indore
16	HI-8802	*Triticum turgidum* ssp. *durum*	IARI, Regional Station, Indore
17	HI-8627	*Triticum turgidum* ssp. *durum*	IARI, Regional Station, Indore
18	HI-8805	*Triticum turgidum* ssp. *durum*	IARI, Regional Station, Indore
19	Local-12	*Triticum aestivum* L.	Local Gahun, Vill.Mohanpurwa, Banda
20	Local-13	*Triticum aestivum* L.	Local Gahun, KVK Jalaun
21	Local-14	*Triticum aestivum* L.	Local Gahun, KVK Jalaun
22	Local-15	*Triticum turgidum* ssp. *durum*	Vill.Panwari, Mahoba
23	Local-16	*Triticum aestivum* L.	Vill.Panwari, Mahoba
24	DBW 187	*Triticum aestivum* L.	IIWBR, Karnal, Haryana
25	Local-17	*Triticum aestivum* L.	Mawai Banda, Local Collection
26	Raj 4120	*Triticum aestivum* L.	IIWBR, Karnal, Haryana
27	Local-18	*Triticum aestivum* L.	MawaiBanda, Local Collection
28	PBW 34	*Triticum turgidum* ssp. *durum*	PAU, Ludhiana Punjab
29	PDW 274	*Triticum turgidum* ssp. *durum*	PAU, Ludhiana Punjab
30	PDW 291	*Triticum turgidum* ssp. *durum*	PAU, Ludhiana Punjab
31	PDW 314	*Triticum turgidum* ssp. *durum*	PAU, Ludhiana Punjab
32	DWL 5023	*Triticum turgidum* ssp. *durum*	PAU, Ludhiana Punjab
33	PDW 215	*Triticum turgidum* ssp. *durum*	PAU, Ludhiana Punjab
34	PDW 233	*Triticum turgidum* ssp. *durum*	PAU, Ludhiana Punjab

**Table 2:** Details of the experiments conducted at BUAT, Banda and KVK, Jhansi during 2020–2021 and 2021–2022 crop seasons.

Locations	Year	Environments	Date of sowing	Date of harvesting	Plant Response
BUAT, Banda	2020–2021 2021–2022	Timely sown, irrigated	05/11/2020	11/03/2021 to 02/04/2021	Optimum environment (OE)
		Late sown, irrigated	15/12/2020	28/03/2021 to 13/04/2021	Heat stress environment (HSE)
		Late sown, restricted irrigation	15/12/2020	22/03/2021 to 07/04/2021	Combined heat–drought stress environment (HDSE)
KVK, Jhansi	2020–2021 2021–2022	Timely sown, irrigated	06/11/2020	14/03/2021 to 01/04/2021	Optimum environment (OE)
		Late sown, irrigated	18/12/2020	27/03/2021 to 13/04/2021	Heat stress environment (HSE)
		Late sown, restricted irrigation	18/12/2020	24/03/2021 to 11/04/2021	Combined heat–drought stress environment (HDSE)

a. Crop Research Farm, Banda University of Agriculture and Technology Banda, UP (25.5269°N latitude and 80.3418°E longitude)

b. Experimental Farm, Krishi Vigyan Kendra, Jhansi (25.5347° N latitude, 78.5742° E longitude)

Bundelkhand region in Uttar Pradesh state of India is known for extreme heat and drought stress, which severely affect the growth, development, and yield of wheat crop. This region frequently experiences extreme temperature, usually 50°C during summer and 5°C in the winter season. The summer season (April–June) characterized by strong heat wave and dusty, gusty, and dry wind blowing over the entire region, which often lead to fatal heat strokes. The average rainfall of this region is 800–900 mm per annum; however, most of rainwater is lost as runoff due to undulated topography. During winter season (November–March), few rain showers are received occasionally, which supplies inadequate moisture to crops. The whole Bundelkhand region is known for predictable drought and heat stress during the anthesis to grain filling stage of winter season crops.

### Experimental design

The experiments were conducted under three environments, *viz*., the optimum environment (OE), heat stress environment (HSE), and the heat–drought environment (HDSE) (**Table 2**). The timely sown (OE) and late sown experiments (HSE) were given irrigation (flood irrigation) at the required field capacity throughout the crop period, whereas in the late sown restricted irrigation, no irrigation was given during the reproductive phase (HDSE). The genotypes were planted in a two-row plot of 2 m length with two replicates following Alpha lattice design for each condition. The distance of 5 m was kept between the trials to check the moisture percolations in the drought experiment. The sowing was done with the modified precision numeric seed planter. The sowing and harvesting dates for each environment for both the years is given in **Table 2**. The crop was supplemented with 120 kg/ha nitrogen and 60 kg/ha phosphorus. Total N was split into three doses of 40 kg/ha each and applied at the three-leaf stage, tillering, and again at heading stage. The recommended agronomic practices of weeding and crop protection were applied to raise healthy crop plants.

### Measurement of phenological and yield-related traits

The data on yield and yield-contributing traits were recorded for each genotype in all three environments during 2020–2021 and 2021–2022.

• Days 50% heading (DH): number of days taken from date of sowing to appearance of anthers on 50% plants in a plot
• Days to maturity (DM): number of days taken from date of sowing until physiological maturity completed
• Numbers of tillers/plant (NTP): actual count of the productive numbers of tillers of five plants (spike bearing) per plant
• Spike length (SL): measured from base of spike to the end tip of spike excluding awns in centimeters.
• Number of spikelets/spike: counting number of spikelets per spike on main spike
• Plant height (PH): taken from base of soil surface to terminal of spike excluding awns in centimeters of 10 plants
• Number of grain/spike (NGS): counting grain numbers in five randomly selected spike and divide by 5
• Test kernel weight (TKW): 100 seeds were randomly taken for each genotype, and weight was recorded in grams
• Biological yield (BY): measured by whole plant weight including shoot and leaves with kernel after drying
• Seed yield per plot (SY): measured in grams using a sensitive balance after the moisture of the seed is adjusted to 12%. Total dry weight of grains harvested from each plot was taken as grain yield per plot
• Harvest index (%): estimated by dividing grain yield per plot to biological yield per plot

#### Calculation of stress tolerance indices

The experiments were conducted under three different environments, *viz*., the optimum environment (OE), heat stress environment (HSE), and the heat–drought environment (HDSE). The selection indices for heat stress and combined heat–drought stress were calculated based on the relationship among yield under normal and stress environment. The stress tolerance indices were calculated as follows:

Stress tolerance index (STI): STI = $\frac{\left(\text{Yp}\right).\left(\text{Ys}\right)}{{\left(\bar{\text{Yp}}\right)}^{2}}$ (Fernandez, 1992)

Stress susceptibility index: SSI = $\frac{1-\left(\frac{\text{Ys}}{\text{Yp}}\right)}{1-\left(\frac{\bar{\text{Ys}}}{\bar{\text{Yp}}}\right)}$ (Fischer and Maurer, 1978)

Mean productivity (MP): MP = $\frac{\left(\text{Yp}+\text{Ys}\right)}{2}$ (Rosielle and Hamblin, 1981)

Tolerance (TOL): TOL = Yp−Ys (Rosielle and Hamblin, 1981)

Modified stress tolerance index (MSTI): MSTI = $\frac{{\left(\text{Ys}\right)}^{2}}{{\left(\bar{\text{Ys}}\right)}^{2}}\times \text{STI }$ (Farshadfar and Sutka, 2002)

where Yp is the yield under optimal conditions, Ys is the yield under stress, $\bar{\text{Yp}}$ is the average yield of all entries under optimal conditions, and $\bar{\text{ Ys}}$ is the average yield of all entries under stress.

MP favors higher yield potential and lower stress tolerance, and the selections based on MP generally increases the average performance of genotypes in both stress and non-stress environments and fails to distinguish between stress tolerant and high yielding genotypes. Larger values of TOL represents more sensitivity to stress; thus, smaller values are favored for selecting tolerant genotypes. The smaller the values of SSI and TOL, the greater the value of tolerance but fails to distinguish between genotypes performing better under both environments and performing better only under stress. Selection based on TOL and SSI favors those genotypes having lower yield potential under normal and high yield potential under stress environments. Thus, TOL also fails to distinguish between tolerant and high yielding genotypes. The STI is used for the identification of wheat genotypes that produce higher yields under non-stressed and stressed environments. The genotypes selected based on STI will have higher stress tolerance and grain yield. Genotypes having higher values of STI are tolerant and have high yield potential, as it involves the stress tolerance intensity and geometric mean productivity (GMP). Thus, STI is the best one to distinguish between genotypes performing superior/poor either under stress or non-stress environments or under both environments.

#### Statistical analysis

The analysis of variance (ANOVA) was performed, first taking locations, years, and environments as random factor. In the second stage, ANOVA was performed location wise separately for all three environments. The analysis of alpha lattice experimental design for all agro-morphological and physiological traits was computed using Agricolae function of R package. The location, year, environment, genotypes, and their interaction, standard errors and least significant difference test (LSD), and Tukey’s honest significant difference (HSD) test were performed. The basic statistical parameters and graphical representation through box plot of data matrix for all studied traits were performed with IBM SPSS software and PAST3 (version 1.0.0.0) to know about the variability in the traits among germplasm. The correlation analysis was conducted for each environment (pooled across years and locations) using correlation plot function of “metan” package (Olivoto and Lúcio, 2020) in R package.

## Results

### Environment assessment

**Figure 1:**
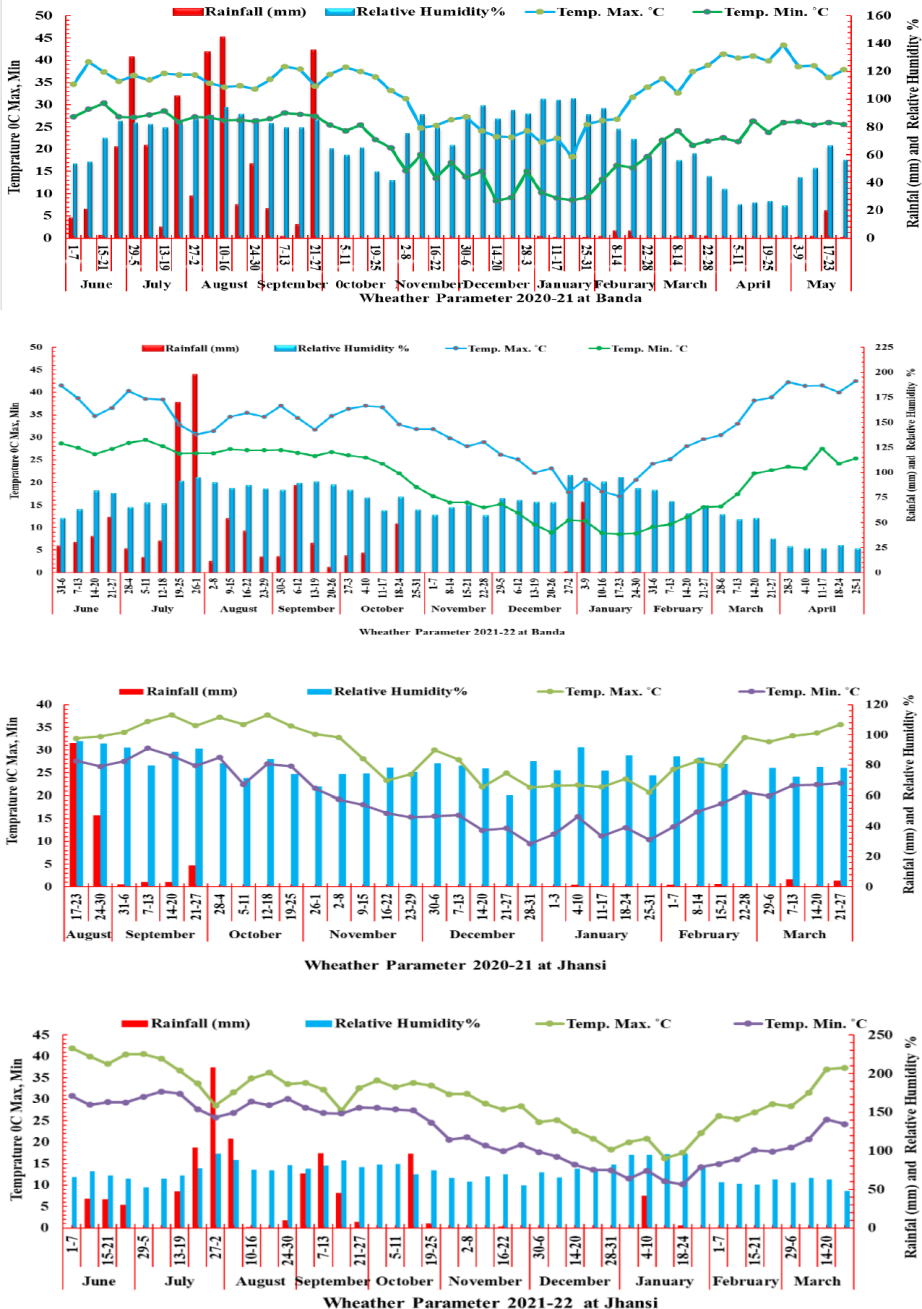
Weather parameter rainfall (mm), temperature °C (maximum and minimum) relative humidity (%) in two growing season 2020–2021 and 2021–2022 at Banda and Jhansi locations.

The Banda and Jhansi locations are typically represented by hot and dry environment of Central Zone (CZ) in India. The weather data were recorded at the Meteorological Observatory Unit (MOU) of the University at Banda and KVK, Jhansi for two years (**Figure 1**). The maximum temperature was recorded more than 35°C at the grain filling stage during 2020–2021 and 2021–2022 at the Banda and Jhansi locations. At Banda location, the cropping season during 2020–2021 was extended due to 10 mm rainfall in the month of March, while during 2021–2022, the temperature in the last week of February increased rapidly exposing the crop to terminal heat. During 2020–2021, maximum precipitation (958 mm) was received from June to October during 2020–2021, while 929 mm of rainfall was recorded in the monsoon season during 2021–2022. During the month of February 2021, very less rainfall of 10.57 mm was received in the month of February 2021, whereas rainfall of 104 mm was received at early vegetative stage of the crop in the first week of January 2022. During 2022, the rainfall was received only in the early vegetative stage, and the crop was exposed to drought stress during reproductive phase. The OE trials were irrigated at both locations to avoid the effect of heat and drought stress. The relative humidity (RH) at the grain filling stage during 2020–2021 was high due to rain in the month of March, while RH was low during 2021–2022 at the time of grain filling.

### Analysis of variance

Combined analysis of variance using linear mixed model (LML) indicated significant effects of environment, location, year, and their interaction on most of the measured traits (**Table 3A**). The genotypes exhibited highly significant variation for all traits except TKW and HI. The pooled analysis of variance indicated significant differences in all interactions; thus, a separate analysis of variance was performed for each location. The individual location ANOVA analysis also indicated significant differences between genotypes, and Gen × Env interaction was also significant for most of the traits (**Tables 3B****,** **C**).

**Table 3A:** Combined analysis of variance of agronomic traits across environment and locations in two growing periods (2020–2021 and 2021–2022).

Response	Df	DFF	DM	PH	NT	SL	NSS	NGS	TKW	BY	HI	SY
Location	1	3,438***	1,233***	11,014 ***	38.6 *	20.99	4.37	66	15,861	31.61 ***	83	11.1***
Year	1	383***	315***	1,831 ***	1,538.2 ***	12.38	83.03***	1,026 ***	10,928	0.97 *	1,363	1.4***
Env	2	14,421***	69,455***	4,578 ***	101.5 ***	7.92	171.68***	6,521 ***	13,363	77.67 ***	8,342	9.5***
Gen	33	115***	64***	5,445 ***	46.6 ***	154.27 ***	7.59***	614 ***	12,401	1.12 ***	3,563	0.2***
Location:Gen	33	8**	11***	122 ***	14 *	11.53	3.76	41	12,529	0.14	3,598	0.04***
Year:Gen	33	9**	10***	180 ***	13	10.93	3.55	43	12,480	0.14	3,279	0.01***
Env:Gen	66	20***	15***	88 ***	12.6 *	61.41	4.37	109 ***	12,477	0.55 ***	3,507	0.05***
Location:Env	2	114***	156***	715 ***	305.1 ***	12.67	19.1**	63	12,467	4.87 ***	10,215 *	0.24***
Location:Year	1	1,248***	879***	24,665 ***	2,723.5 ***	297.06 *	14.69*	2,308 ***	11,556	3.62 ***	12,550	0.12***
Year:Env	2	96***	407***	521 ***	1.8	4.31	11.68*	55	13,002	1.8 ***	4,293	0.26***
Location:Year:Gen	33	53***	18***	144 ***	14.7 *	111.47 ***	5.15*	155 ***	12,469	0.13	3,026	0.01***
Location:Env:Gen	66	6	3	73 **	13.1 *	11.52	3.39	36	12,486	0.1	3,324	0.02***
Locat:Year:Env:Gen	66	15***	12***	96 ***	11.1	65.17 *	4.39.	69	12,494	0.11	3,093	0.01***
Replication	1	15	1	170	0.1	14.53	40.37***	380 *	12,289	0.43	2,314	0.08***
Block(Replication)	2	18*	9	3	5.6	149.98 *	15.11*	474 ***	13,354	0.09	42	0.01
Residuals	405	5	4	0.01	8.9	46.32	3.49	63	12,488	0.17	3,321	0.01
Heritability		87	85	98	75	94	45	88	95	83	92	92

*,**,*** significant at alpha 0.05, 0.01 and 0.001 respectively.

Gen, genotype; Env, environment; year, growing season; DFF, day to 50% flowering (number of days); DM, days to maturity (number of days); PH, plant height (cm); NT, number of tillers per plant (number); SL, spike length (cm); NSS, number of spikelets per spike; NGS, number of grains per spike; TKW, test kernel weight (g); BY, biological yield (g); HI, harvest index; SY, seed yield (g/plot).

**Table 3B:** Analysis of variance of agronomic trait across environments at Banda location.

Response	Df	DFF	DM	PH	NT	SL	NSS	NGS	TKW	BY	HI	SY
**Year**	1	124.1**	1,123.4**	6,527.3**	76.1**	44.5**	83.8**	54.6	64.4**	418,048.0	2873.6**	368,641.4**
**Env**	2	6,779.0**	36,225.6**	4,380.8**	372.4**	25.6**	68.4**	2,306.6**	8.9**	24,147,869.6**	1023.5**	4,250,351.2**
**Gen**	33	67.9**	51.2**	3,113.9**	17.1**	11.5**	2.8**	391.3**	2.0**	441,384.1**	746.4**	233,916.3**
**Year:Gen**	33	23.8**	10.7**	134.9**	4.9**	1.8**	1.5**	62.8	0.2	237,276.9*	72.0*	19,043.2**
***Env:Gen***	66	18.9**	11.7**	49.3**	6.0**	1.2**	1.9**	65.3	0.3**	208,553.7*	48.8	46,404.8**
**Year:Env:Gen**	368	15.7**	49.5**	112.6**	7.0**	1.0*	1.6**	40.5	0.4**	241,299.8**	123.1**	37,696.9**
**Rep**	1	11.7	0.1	60.7	8.3	0.1	2.2	60.2	0.0	20,584.3	74.8	62,902.8
**Block(Rep)**	2	8.1	0.4	41.1	2.3	0.8	0.8	209.8	0.2	88,019.9	0.5	3,389.8
**Residuals**	201	4.55	3.3	17.4	2.1	0.7	0.8	53.8	0.2	146,989.0	43.6	7,090.5

*,** significant at alpha 0.05 and 0.01 respectively.

Gen, genotype; Env, environment; year, growing season; DFF, day to 50% flowering (number of days); DM, days to maturity (number of days); PH, plant height (cm); NT, number of tillers per plant (number); SL, spike length (cm); NSS, number of spikelets per spike; NGS, number of grains per spike; TKW, test kernel weight (g); BY, biological yield (g); HI, harvest index; SY, seed yield (g/plot).

**Table 3C:** Analysis of variance of agronomic trait across environments at Jhansi location.

Response	Df	DFF	DM	PH	NT	SL	NSS	NGS	TKW	BY	HI	SY
**Year**	1	1,506.5**	90.4**	19,969.6**	1,637.0**	8.9**	0.6	2,059.2**	4.4**	3,598,135.4**	1183.7**	1,207,070.8**
**Env**	2	7,755.9**	33,148.3**	912.7**	39.0**	10.0**	10.1**	3,110.3**	9.8**	56,646,393.1**	2063.2**	5,560,100.0**
**Gen**	33	55.2**	20.8**	2,453.7**	14.8**	10.4**	2.5*	190.2**	2.6**	801,923.0**	642.2**	97,075.9**
**Year:Gen**	33	38.1**	15.2**	188.3**	9.8**	1.5	1.8	98.1*	0.0	28,753.0	40.8	8,770.1**
***Env:Gen***	66	6.7*	6.9*	111.2*	7.0**	0.9	1.9	54.2	0.2**	441,967.2**	201.2**	31,537.1**
**Year:Env:Gen**	368	16.6**	92.8**	138.0**	7.7**	1.4	3.4**	52.3	0.1**	44,989.7	45.8	1,958.1
**Rep**	1	3.9	2.5	113.3	0.3	0.3	0.0	217.1	2.0**	689,871.9	426.9*	26,626.5**
**Block(Rep)**	2	9.9	16.3*	62.5	9.7	0.7	6.7**	286.6	0.0	15,416.9	158.9	4,374.3
**Residuals**	201	4.8	4.7	77.1	4.2	1.2	1.5	57.6	0.1	193,719.4	80.1	1,965.0

*,** significant at alpha 0.05 and 0.01 respectively.

Gen, genotype; Env, environment; year, growing season; DFF, day to 50% flowering (number of days); DM, days to maturity (number of days); PH, plant height (cm); NT, number of tillers per plant (number); SL, spike length (cm); NSS, number of spikelets per spike; NGS, number of grains per spike; TKW, test kernel weight (g); BY, biological yield (g); HI, harvest index; SY, seed yield (g/plot).

### Effects of terminal heat and combined heat–drought stress on yield and yield components

**Figure 2:**
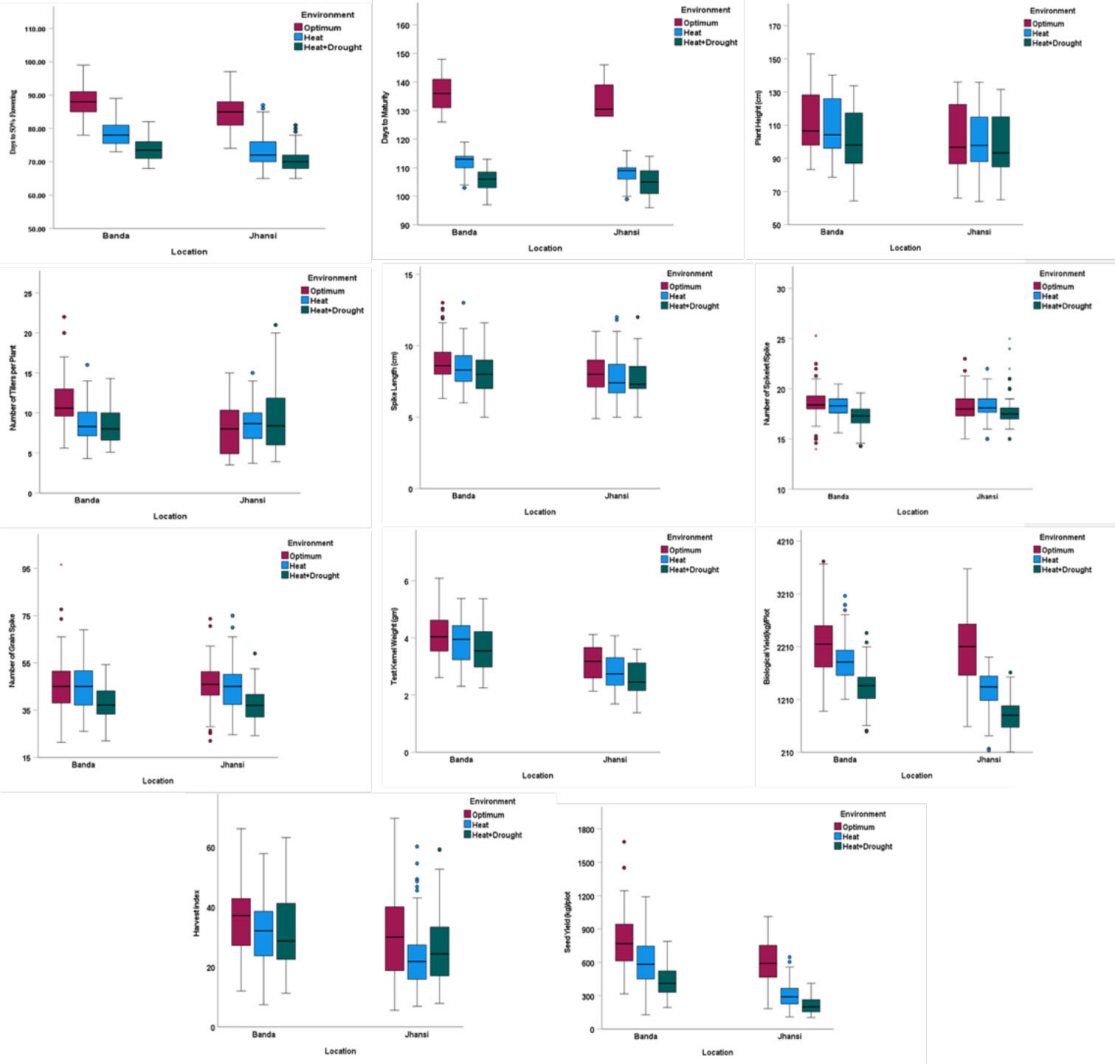
Performance of wheat genotypes for different agronomic traits DFF, day to 50% flowering (number of days); DM, days to maturity (number of days); PH, plant height (cm); NT, number of tillers per plant (number); SL, spike length (cm); NSS, number of spikelets per spike; NGS, number of grains per spike; TKW, test kernel weight (g); BY, biological yield (g); HI, harvest index; SY g/plot, seed yield (g/plot) under all three environments at Banda and Jhansi locations.

Phenotypic trait variation was observed across 34 genotypes under OE, HSE, and HDSE, indicating considerable genetic variation in performance across environments. Some genotypes exhibited higher reduction, while other genotypes managed to grow and remain relatively healthy. The performance of genotypes under combined heat–drought stress was severely affected compared to heat stress alone at Banda and Jhansi. The mean of 11 agro-morphological traits of the wheat lines under optimal and stress conditions is presented in **Table 4** and **Figure 2**. The highest heritability was reported for PH (98%) followed by TKW (95%), whereas least was reported for NSS (45%).

**Table 4:** Basic statistics of agronomic traits under optimum, heat stress, and heat–drought stress environments at Banda and Jhansi for two consecutive growing seasons.

Banda location												
Trait	Optimum environment				Heat stress environment				Heat–drought stress environment			
	Min.	Max.	Mean	Std. Deviation	Min.	Max.	Mean	Std. Deviation	Min.	Max.	Mean	Std. Deviation
DFF	78	99	88	4.7	73	89	79	3.9	68	82	74	3.4
DM	126	148	137	6.1	103	119	112	3.0	97	113	106	3.6
PH	83	153	112	17.7	79	140	109	16.8	64	134	101	18.9
NT	6	17	11	2.5	4	16	8.69	2.2	5	14	8	2.1
SL	6	13	9	1.4	6	13	9	1.3	5	12	8	1.4
NSS	14	25	19	1.7	16	21	18	1.0	14	20	17	1.0
NGS	21	78	45	10.2	26	69	45	9.8	22	54	38	7.0
TKW	3	6	4	0.7	2	5	4	0.8	2	5	4	0.7
BY	985	3,825	2,277	592.4	1,213	3,170	1,942	372.2	602	2,470	1,440	351.2
HI	12	66	36	12.4	7	58	32	10.1	11	63	31	10.9
SY	403	1,279	790	242.4	265	1045	634	205.1	205	985	512	128.5
Jhansi location												
	Optimum environment				Heat stress environment				Heat–drought stress environment			
Trait	Min.	Max.	Mean	Std. Deviation	Min.	Max.	Mean	Std. Deviation	Min.	Max.	Mean	Std. Deviation
DFF	74	97	85	4.7	65	87	73	4.5	65	81	70	3.3
DM	128	146	134	6.1	99	116	108	3.2	96	114	105	4.4
PH	66	136	103	19.2	64	136	100	18.4	65	132	97	18.1
NT	4	15	8	3.4	4	15	9	2.2	4	21	9	3.9
SL	5	11	8	1.4	5	12	8	1.5	5	12	8	1.3
NSS	15	23	18	1.3	15	22	18	1.3	15	22	18	1.6
NGS	22	74	46	8.8	25	75	44	10.6	24	59	37	6.4
TKW	2	4	3	0.6	2	4	3	0.6	1	4	3	0.5
BY	695	3,685	2193	725.3	245	2,013	1,392	361.1	215	1,720	916	325.2
HI	6	70	31	14.4	7	60	23	9.7	8	59	26	11.0
SY	250	930	598	194.2	175	680	361	106.6	141	490	243	73.0

DFF, day to 50% flowering (number of days); DM, days to maturity (number of days); PH, plant height (cm); NT, number of tillers per plant (number); SL, spike length (cm); NSS, number of spikelets per spike; NGS, number of grains per spike; TKW, test kernel weight (g); BY, biological yield (g); HI, harvest index; SY, seed yield (g/plot).

### Impact of heat stress on agro-morphological traits

Heat stress significantly affected the performance of all the traits. At Banda location, highest reduction was reported in NT (24%) followed by SY (20%), DM (18%), and BY (16%) as compared to optimum environment, whereas least reduction of 4% was reported in PH and NSS (**Table 5**). Similarly at Jhansi, highest reduction was reported in SY (44%) followed by (34%), and DM (22%) as compared to optimum environment, whereas least reduction of<5% was reported in PH and NSS (**Table 5**). The maximum yield reduction of 48% was recorded in genotype PDW-233 at Banda, whereas yield reduction of 66% was reported in Local-5 at Jhansi location. The genotype Local-4 recorded minimum SY reduction of 5.6% at Banda, while a minimum 15% SY reduction was recorded in the HI 8627 genotype at Jhansi.

**Table 5:** Percent reduction in yield components of bread wheat under heat stress and combined heat–drought stress environment at Banda and Jhansi location.

Reduction % OE-HSE (Banda)				Reduction % OE-HDSE (Banda)		
Trait	Min	Max	Mean	Min	Max	Mean
**DFF**	1.0	19.6	10	6.34	23.1	16
**DM**	15.1	22.0	18	19.4	27.2	23
**PH**	0.1	10.1	4	1.7	17.9	10
**NT**	4.2	47.6	24	4.3	52.1	27
**SL**	0.7	18.2	7	5.6	32.1	18
**NSS**	0.2	10.7	4	1.3	14.3	8
**NGS**	0.9	20.6	7	1.9	35.1	21
**TKW**	0.3	19.0	8	0.7	26.8	13
**BY**	0.4	37.6	16	16.4	57.0	37
**HI**	0.5	47.6	14	0.1	34.4	15
**SY**	5.6	48.0	20	19.0	65.1	36
Reduction % OE-HSE (Jhansi)				Reduction % OE-HDSE(Jhansi)		
Trait	Min	Max	Mean	Min	Max	Mean
**DFF**	8.2	20.4	14	12.6	22.4	17
**DM**	16.6	25.8	22	19.9	27.1	25
**PH**	0.2	16.5	5	2.7	13.0	8
**NT**	0.3	34.9	10	2.1	35.7	16
**SL**	1.2	20.8	7	0.5	21.1	9
**NSS**	0.1	12.0	3	0.6	18.7	7
**NGS**	0.2	36.8	11	6.1	35.9	21
**TKW**	1.2	19.7	9	2.7	40.8	19
**BY**	1.3	66.0	34	30.3	85.9	57
**HI**	0.2	60.4	24	0.4	61.4	21
**SY**	15.0	66.0	44	35.4	79.2	59

DFF, day to 50% flowering (number of days); DM, days to maturity (number of days); PH, plant height (cm); NT, number of tillers per plant (number); SL, spike length (cm); NSS, number of spikelets per spike; NGS, number of grains per spike; TKW, test kernel weight (g); BY, biological yield (g); HI, harvest index; SY, seed yield (g/plot).

### Impact of combined heat–drought stress on agro-morphological traits

Combined heat–drought stress also affected agro-morphological traits across years at both locations. At Banda, highest reduction was recorded for BY (37%) followed by SY (36%) and NT (27%), whereas the least affected trait was NSS, which showed a reduction of 8% under combined heat–drought stress. Spike yield (59%) showed the highest reduction at Jhansi followed by (57%) and DM (25%), whereas NSS recorded the minimum reduction of 7% (**Table 5**). The PH and NSS traits were the least affected traits under heat–drought stress at both locations. Highest SY was reported for genotype Local-17 (985 g) at Banda, whereas the genotype PDW-274 recorded with a maximum SY of 490 g at Jhansi. Genotype Local-3 and HI8627 recorded minimum SY reduction of 19% and 35% at Banda and Jhansi, respectively. Under HDSE, DFF significantly reduced from 88 days to 74 days. Genotypes Local-17 and Local-13 were earliest in heading at Banda, whereas genotypes Local-3, Local-9, HI-8777, Local-13, Local-14, and DBW 187 were earliest in heading at the Jhansi.

Among all genotypes, Local-17 and HI 8802 performed well under HSE and HDSE with higher yield at the Banda location. At Jhansi, genotypes DBW 187 and HI 8777 performed well under HSE with higher yield, while under HDSE genotype, PDW 274 and HI 8777 were better performing genotypes.

#### Correlations analysis

Correlation analysis was undertaken to assess the association of traits under normal and stress environments using pooled environment wise data (**Tables 6A, B**). The SY showed significant positive correlation with NGS (0.73**) and HI (0.78**), whereas it showed significantly negative correlation with NT and PH under optimum environment. Under heat stress environment, SY showed significantly positive correlation with HI (0.89**), NGS (0.80**), NSS (0.49**), and TKW (0.66**). In contrast to this, in the combined heat–drought environment, SY indicated significant correlation with BY (0.37**), NGS (0.68**), NSS (0.37**), HI (0.42*), and TKW (0.38**). There was a significantly negative correlation of PH (−0.44*) and NT (−0.61) under HSE, whereas SY had a significantly negative correlation with DFF (−0.53**) and DM (−0.61**). NGS, NSS, and TKW are the main traits contributing towards yield under stress environments, whereas BY is a key trait to be considered under combined heat and drought stress.

**Table 6:** Correlation analysis between different agro-morphological traits based on pooled data across different environments.

a. Correlation analysis under optimum environment											
	DFF	DM	HI	NGS	NSS	NT	PH	SL	SY	TKW	
**BY**	0.12	0.12	-0.7	-0.17	0.17	0.33	0.47*	0.34	-0.16	-0.48**	
**DFF**		0.36*	-0.12	-0.11	0.05	0.02	-0.22	-0.28	-0.04	-0.02	
**DM**			0.01	-0.17	-0.13	0.1	-0.19	-0.43*	0.06	0.2	
**HI**				0.59**	0.11	-0.56**	-0.66**	-0.35*	0.78**	0.75**	
**NGS**					0.45*	-0.31	-0.19	0.19	0.73**	0.28	
**NSS**						0.18	0.1	0.42*	0.34	-0.03	
**NT**							0.53**	0.31	-0.45*	-0.54**	
**PH**								0.61**	-0.51**	-0.53**	
**SL**									-0.15	-0.49**	
**SY**										0.64**	
b. Correlation analysis under heat stress (above diagonal) and combined heat-drought (below diagonal) environments											
	BY	DFF	DM	HI	NGS	NSS	NT	PH	SL	SY	TKW
**BY**		-0.28	-0.29	-0.37*	0.09	0.32	0.07	0.45*	0.46*	0.07	-0.19
**DFF**	-0.50**		0.52**	-0.17	-0.29	-0.42*	-0.05	-0.36*	-0.42*	-0.34	-0.13
**DM**	-0.43*	0.46*		-0.18	-0.15	-0.40*	0.01	-0.29	-0.44*	-0.32	-0.09
**HI**	-0.64**	0.1	0.01		0.68**	0.33	-0.60**	-0.62**	-0.41*	0.89**	0.72**
**NGS**	0	-0.14	-0.3	0.51**		0.42*	-0.53**	-0.44*	-0.1	0.80**	0.48**
**NSS**	0.08	-0.08	-0.3	0.19	0.35*		-0.04	-0.05	0.34*	0.49**	0.11
**NT**	0.49**	-0.16	-0.04	-0.74**	-0.61**	-0.22		0.52**	0.40*	-0.61**	-0.62**
**PH**	0.74**	-0.28	-0.23	-0.76**	-0.45*	-0.26	0.74**		0.47*	-0.44*	-0.44*
**SL**	0.64**	-0.45*	-0.31	-0.51**	-0.17	0.12	0.29	0.58**		-0.22	-0.46*
**SY**	0.37*	-0.53**	-0.61**	0.42*	0.68**	0.37*	-0.31	-0.11	0.12		0.66*
**TKW**	-0.3	0.14	-0.07	0.49**	0.55**	0.18	-0.55**	-0.57**	-0.42*	0.38*	

*, ** significant at alpha 0.05 and 0.01 respectively.

**Figure 3:**
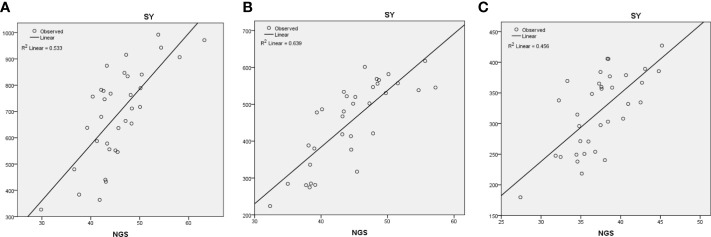
Regression analysis of seed yield (SY) with number of grains/spike (NGS) under **(A)** optimum, **(B)** heat stress, and **(C)** combined heat–drought stress environments.

The regression analysis was also undertaken to assess the contribution of different traits towards final grain yield. Under optimum environment, NGS (R^2^ = 0.53) followed by TKW (R^2^ = 0.20), PH (R^2^ = 0.06), and BY (R^2^ = 0.03) contributed significantly towards SY (**Figure 3A**). Under HSE, NGS (R^2^ = 0.63) contributed more towards SY followed by TKW (R^2^ = 0.09) and NSS (R^2^ = 0.04) (**Figure 3B**), whereas under HDSE, NGS (R^2^ = 0.46) contributed highest followed by DFF (R^2^ = 0.19) and DM (R^2^ = 0.07) (**Figure 3C**).

### Identification of heat-tolerant genotypes

Stress indices (STI, SSI, MP, TOL, and MSTI) were estimated based on seed yield under stress environments (HSE and HDSE) and OE conditions. Based on the STI and SSI, the genotypes were grouped into tolerant and sensitive to heat and combined drought-heat stress conditions (**Supplementary Tables S1**–**S4**).

### Heat-tolerant genotypes

Based on STI, five genotypes local-17 (2.16) and PDW 274 (1.88), HI-8802 (1.83), HI-8713 (1.54), and Local-5 (1.32) performed better under heat stress environment at Banda (**Table 7**). Similarly, five genotypes DBW 187 (1.77), HI-8777 (1.69), Local-4 (1.53), Raj 4120 (1.48), and PDW 274 (1.40) were tolerant at Jhansi (**Table 7**). Thus, these genotypes are considered as heat stress tolerant. Based on SSI, genotypes PDW 233 (2.05) and Local-16 (1.96) were identified as heat susceptible at Banda (**Supplementary Table S1**), whereas at Jhansi, the maximum value of SSI was exhibited in Local-5 (1.34) and PDW 233 (1.33) (**Supplementary Table S3**); thus, these genotypes are considered as heat stress susceptible. The maximum value of TOL was recorded in PDW 233 (437) and Local-16 (365) in the HSE at Banda (**Supplementary Table S1**), whereas genotypes Local-5 (522) and PDW 233 (516) had a maximum value of TOL under HSE at Jhansi (**Supplementary Table S3**).

**Table 7:** Seed yield and tolerance indices of five highly heat tolerant genotypes of wheat at Banda and Jhansi. based on mean of two years data.

Location	S.No.	Cultivar Name	SY(g)	STI	SSI	MP	TOL	MSTI
Banda	1	Local-17	1,279	2.16	0.78	1,801	233	6.5
	2	PDW 274	1,165	1.88	0.61	1,665	166	5.2
	3	HI-8802	1,118	1.83	0.41	1,625	107	5.2
	4	HI-8713	1,035	1.54	0.47	1,496	113	3.6
	5	Local-5	957	1.32	0.45	1,385	12	2.7
	Mean		1,111	1.75	0.54	1,594	144	4.6
Jhansi	1	DBW 187	930	1.77	0.54	1,270	250	8.9
	2	HI-8777	895	1.69	0.49	1,233	219	8.5
	3	Local-4	839	1.53	0.45	1,165	187	7.1
	4	Raj 4120	815	1.48	0.41	1,139	166	6.8
	5	PDW 274	820	1.40	0.52	1,125	210	5.7
	Mean		860	1.57	0.48	1,186	206	7.4

### Heat–drought-tolerant genotypes

Under HSDE, highest values for STI were recorded in genotypes Local-17, PDW 274, HI-8802, HI-8713, and HI-8759 at Banda (**Table 8**). At Jhansi, genotypes PDW 274, DBW 187, HI-8777, Raj 4120, and Local-4 had the highest value of STI under HDSE. Hence, these genotypes can be considered as heat–drought stress tolerant. Based on SSI, the higher value was recorded in genotypes PDW 233 and PDW 291 at Banda (**Supplementary Table S2**), whereas at Jhansi, genotypes PDW 233 and DWL 5023 recorded the highest values (**Supplementary Table S4**). The maximum values of TOL recorded were in genotypes PDW 233 and PDW 291 at Banda, whereas genotypes PDW 233 and Local-5 recorded maximum values at Jhansi.

**Table 8:** Seed yield and stress tolerance indices of five highly heat–drought-tolerant genotypes of wheat at Banda and Jhansi based on mean of two years data.

Location	S.No.	Cultivar Name	SY(g)	STI	SSI	MP	TOL	MSTI
Banda	1	Local-17	1,279	2.04	0.50	1,771	293	10.8
	2	PDW 274	1,165	1.64	0.55	1,602	292	6.8
	3	HI-8802	1,118	1.62	0.43	1,567	221	7.2
	4	HI-8713	1,035	1.26	0.60	1,410	285	3.9
	5	HI-8759	983	1.09	0.66	1,327	295	2.8
	Mean		1,116	1.53	0.55	1,536	277	6.3
Jhansi	1	PDW 274	820	1.12	0.62	1,064	330	6.0
	2	DBW 187	930	1.12	0.83	1,145	501	4.6
	3	HI-8777	895	1.08	0.80	1,111	463	4.5
	4	Raj 4120	815	0.95	0.76	1,022	400	3.6
	5	Local-4	839	0.72	0.98	993	532	1.5
	Mean		860	1.00	0.80	1,067	445	4.1

In summary, genotypes Local-17 and PDW 274 were selected to be heat and combined heat and drought tolerant along with yield at Banda. Similarly, genotypes DBW 187, HI 8777, and PDW 274 performed better at Jhansi under both environments compared to the rest of the genotypes.

## Discussion

The confounding effect of heat along with drought stress are more detrimental to plant performance than any individual stress (Barnabas et al., 2008; Li et al., 2013). In the present study, 34 wheat genotypes were evaluated at unexplored field screening sites Banda and Jhansi for heat and combined heat–drought stress during 2020–2021 and 2021–2022. The study aims to quantify the effects of stress on phenological traits development and yield loss. The delayed sowing and restricted irrigation are efficient strategies to identify heat- and drought-tolerant genotypes in a number of crops like bread wheat (Qaseem et al., 2019; Hussain et al., 2021; Yashavanthakumar et al., 2021), Durum wheat (Aberkane et al., 2021; Ayed et al., 2021; Laddomada et al., 2021), mustard (Limbalkar et al., 2021), lentil (Sellami et al., 2021; El haddad et al., 2020; Choudhury et al., 2012), and chickpea (Krishnamurthy et al., 2011; Devasirvatham et al., 2015). The results demonstrated that high temperature coupled with water stress is more destructive to the plant development process and, finally, reflected in yield reduction. The heat and drought stress in combination is more pronounced at Jhansi as compared to that at Banda location.

### Effect of stress on plant phenology and grain yield

The weather conditions of the Bundelkhand region are hot and dry during the reproductive phase for the cool season crops like wheat. The temperature and rainfall pattern are consistently favoring experiments focused on screening germplasm for abiotic stresses, more specifically heat and drought stress. The meteorological data clearly demonstrate that more than 95% rainfall was received from June to September in both cropping seasons at Banda. Similarly, at the Jhansi location during monsoon season, the major part of the total rainfall was received. Therefore, at both locations, weather conditions were ideal for challenging plants to stress conditions (**Figure 1**). During crop season, very less amount of precipitation was received in the reproductive phase, while crop water requirement in the vegetative phase was maintained by irrigation. The temperature in HSE and HDSE experiments was >35°C during the anthesis period, and it reached up to 40°C during the grain filling stage, although experiments under optimum environments were raised under non-stressed conditions.

Our result showed that combined stress penalty on seed yield at Jhansi was more severe (59% under HDSE as compared to 44% under HSE). The maximum yield penalty (36%) was recorded in HDSE, whereas yield penalty of 20% was estimated under heat stress at Banda location (**Table 4**). The maximum yield for each genotype was harvested at Banda due to the long duration of crop compared to Jhansi. The climatic conditions particularly rainfall and temperature patterns and recorded yield penalty due to stress environments at both locations reflect that Bundelkhand is an appropriate location for terminal heat and drought stress evaluation.

The combined stress is more detrimental on plant phenological traits than heat stress alone at both the locations (**Table 5**). Several reports have shown that the combination of heat and drought stress is common in dry and semi-dry regions worldwide and reported extensive yield losses. The results of the present study are in agreement with the study of Yashavanthakumar et al. (2021), which reported the confounding effect of heat and drought resulting in yield loss (56%), followed by drought (41.1%) and least affected by heat stress alone (4.8%) over the control environment. Results of the present study also showed that heat and heat–drought stress adversely affected all measured trait significantly, which is also reported in other wheat crop experiments (Hussain et al., 2021; Yashavanthakumar et al., 2021) and Durum wheat (Aberkane et al., 2021; Ayed et al., 2021; Laddomada et al., 2021). Drought stress at pre- and post-anthesis stage significantly reduced grain yield (Y), spike length (SL), number of grains spikes-1 (NGS) and thousand kernel weight (TKW), while kernel abortion (KA) was increased (Shokat et al., 2020). Our study is in agreement with Qaseem et al. (2019), who reported that the combined heat and drought stress is a more devastating factor to plant development as compared to any stress alone. However, there was less impact of stress conditions on PH and NSS compared to optimum environments. Although, both traits are highly stable and were less affected by the environmental factors. This finding is contrary to previous studies, which have suggested that stress has a significant impact on plant height reduction (Sattar et al., 2020). In conclusion, the genotypes Local-17, PDW 274, HI 8802, and HI 8713 have the highest yield potential under OE at the Banda location, whereas DBW 187, HI 8777, Local-4, and PDW 274 were superior at Jhansi location.

### Correlation among traits

The association among all measured agro-morphological traits with seed yield was studied using pooled data over environments under OE, HSE, and HSDE (**Tables 6A, B**). The NGS and TKW directly positively associated with seed yield across the normal and stress environments, indicating that the genotypes were able to maintain both the traits under stress conditions. The selection of the genotypes under stress conditions is most reliable taking NGS and TKW as targeted traits (Dodig et al., 2012; Sareen et al., 2014). There was a significantly negative correlation of PH (−0.44*) and NT (−0.61) with SY under HSE, whereas SY had a significantly negative correlation with DFF (−0.53**) and DM (−0.61**). The traits DFF and DM indicated that heat and drought escape mechanisms as short duration genotype are preferred for drought and high-temperature tolerance (Reynolds et al., 2007; Punia et al., 2011; Mondal et al., 2013; Gupta et al., 2020). The days to maturity and early heading escapes the stress during the critical reproductive phase and increases productivity before the onset of heat and drought stress (Blum, 2010). NGS, NSS, and TKW are the main traits contributing towards yield under stress environments, whereas BY is a key trait to be considered under combined heat and drought stress.

The regression analysis indicated that NGS is the key trait that is contributing towards grain yield under stress environments (**Figure 3**). High temperature reduces grain weight (Wardlaw et al., 1980) due to reduction in both duration and rate of grain filling and high respiration rate (Tashiro and Wardlaw, 1990); however, yield reduction is primarily due to reduction in grain weight (Guttieri et al., 2001). Tyagi et al. (2003) also reported that grain weight per spike could be used as a measure of heat tolerance. Similarly in the present study, TKW is found to be significantly contributing towards SY under heat stress.

### Selecting stress-tolerant genotypes

In the present study, analysis of variance revealed the significant difference among 34 wheat genotypes for all measured traits. The significant variation in genotype, environment, year, and location indicated variable performance of genotypes across environments. The genotype × environment interaction for SY and its associated trait shows the possibilities of selecting genotypes with less yield penalty under stress environments. The significant effect of different environments on the performance of genotypes was reported by Mohammadi et al. (2010) and Talebi et al. (2010) in bread wheat.

Stress selection indices STI, SSI, MP, TOL and MSTI, are widely used for the identification of tolerant genotypes under stress environments. The selection of heat- and drought-stress tolerant genotypes was done using stress tolerance index in wheat was adopted in several studies (Puri et al., 2015; Aberkane et al., 2021a; Poudel et al., 2021). The STI is a more appropriate index to identify tolerant genotypes in the panel of germplasm. Earlier many researchers used STI to screen germplasm in chickpeas (Erdemci, 2018; Shabani et al., 2018). The genotype having higher values of STI, MP, and MSTI indicates their superior performance under both normal and stress environments. Additionally, the genotypes selected based on STI will have higher stress tolerance and grain yield, as it involves the stress tolerance intensity and GMP.

MP favors higher yield potential and lower stress tolerance, and the selections based on MP generally increase the average performance of genotypes in both stress and non-stress environments and fails to distinguish between stress-tolerant and high-yielding genotypes.

Larger values of TOL represent more sensitivity to stress; thus, smaller values are favored for selecting tolerant genotypes. The smaller the values of SSI and TOL, the greater the value of tolerance but fails to distinguish between genotypes performing better under both environments and performing better only in under stress. Selection based on TOL and SSI favors those genotypes having lower yield potential under normal and high yield potential under stress environments. Thus, TOL also fails to distinguish between tolerant and high-yielding genotypes.

The STI is used for the identification of wheat genotypes that produce higher yields under non-stressed and stressed environments. Genotypes having higher values of STI are tolerant and have high yield potential, as it involves the stress tolerance intensity and GMP. Thus, STI is the best one to distinguish between genotypes performing superior/poor either under stress or non-stress environments or under both environments.

In our screening experiments under HSE and HDSE at Banda, genotypes Local-17, PDW 274, HI 8802, and HI 8713 have the highest values of STI, MP, and MSTI; therefore, these are considered as tolerant to heat and combined heat–drought stress (**Table 7**). Similarly at Jhansi, DBW 187, HI 8777, Local-4, Raj 4120, and PDW 274 showed the highest value for STI, MP, MSI, and MSTI under HSE and HDSE. Our result is in support of the finding of Kamrani et al. (2017) for the selection of higher-yielding and heat tolerant genotypes based on MP and STI. The genotype having a higher value of TOL and SSI indicates more reduction in yield under HSE and HDSE, thus indicating stress susceptibility (**Tables 7**, **8**) (Nouri et al., 2011; Poudel et al., 2021). The smaller the values of SSI and TOL, the greater the value of tolerance but fails to distinguish between genotypes performing better under both environments and performing better only in under stress. The selection based on TOL and SSI favors those genotypes having lower yield potential under normal and stress environments. The susceptibility indices TOL and SSI were less effective than tolerance indices STI, MP, and MSTI to select suitable lines for drought stress (Kamrani et al., 2017).

In our study, genotype PDW 274 showed higher values of stress indices STI, MP, and MSTI and ranked in the top 5 genotypes across the environment at both locations (**Tables 6**, **7**). The other genotypes PDW 233 and PDW 291 showed the highest stress susceptibility index (SSI) across the environments at both locations. The reliability of STI for the selection of stress-tolerant genotypes with high seed yield under normal and stressed conditions further confirms the consistency of this index in selecting for high productivity under stress conditions (Fernandez, 1992).

## Conclusion

In conclusion, high genetic variation was found for agro-morphological traits in a collection of wheat lines cultivated across the Bundelkhand region of Uttar Pradesh. This means that the landraces and recently released cultivars for commercial cultivation had good potential to harness in breeding for heat and drought stress. The selection based on stress indices identified tolerant lines, which could be used to improve stress tolerance in wheat. The stable traits NGS and TKW have shown positive association with SY across environments. Genotypes Local-17, PDW 274, HI-8802 and HI-8713, DBW 187, HI-8777, and Raj 4120 are identified as stress tolerant (heat and drought) at both the locations. The combination of selection indices and consistent association with physiological traits showed the potential to increase the selection efficiency of superior genotypes.

## Data availability statement

The original contributions presented in the study are included in the article/**Supplementary Material**. Further inquiries can be directed to the corresponding author/s.

## Author contributions

Conceptualization: HK, CS and MK. Methodology: HK. Investigation: ShK, SK and AK performed multi-year-location field experiments. Data curation: ShK. Statistical analysis: RK, VG, ShK, HK, and CS. Validation: HK and VG. Resources: MK and GP. Writing—original draft preparation: HK. Review and editing: HK, VG, SK, AS and RK. Visualization: RK. All authors contributed to the article and approved the submitted version.
