# Supplementary material for: Capturing agro-morphological variability for tolerance to terminal heat and combined heat–drought stress in landraces and elite cultivar collection of wheat
Source: Front Plant Sci. 2023 May 9;14:1136455. doi: 10.3389/fpls.2023.1136455 (PMC10214469; doi:10.3389/fpls.2023.1136455)
Supplement: Supplementary file 1 [file Table_1.pdf]

Supplementary Tables

Table S1. Stress indices of 34 genotypes under OE and HSE conditions at Banda

Genotype	STI	SSI	MP	TOL	MSTI
Local-1	0.28	1.18	664.63	134.41	0.10
Local-2	0.36	1.20	756.93	156.21	0.16
Local-3	0.30	0.54	661.18	57.83	0.13
Local-4	1.05	0.24	1220.95	46.04	1.77
Local-5	1.32	0.45	1385.24	101.59	2.67
Local-6	0.94	0.50	1167.30	94.46	1.32
Local-7	0.52	0.77	881.30	112.96	0.37
Local-8	0.72	0.76	1036.22	131.38	0.72
Local-9	0.43	1.55	854.82	234.54	0.20
Local-10	0.33	0.55	699.02	62.54	0.17
Local-11	0.24	0.48	596.05	46.71	0.09
HI-8759	1.28	0.78	1384.65	179.51	2.27
HI-8737	0.69	0.67	1014.97	111.73	0.69
HI-8713	1.54	0.47	1496.26	112.91	3.62
HI-8777	1.26	0.48	1351.89	105.91	2.39
HI-8802	1.83	0.41	1624.61	106.61	5.16
HI-8627	1.30	0.33	1361.64	71.64	2.64
HI-8805	0.90	1.13	1187.51	230.16	1.01
Local-12	0.49	0.71	855.01	100.16	0.34
Local-13	1.24	0.43	1337.81	92.30	2.35
Local-14	0.72	0.84	1042.45	146.54	0.71
Local-15	0.17	1.46	536.05	138.21	0.03
Local-16	0.55	1.96	1009.38	365.16	0.28
DBW 187	1.24	0.69	1357.22	153.74	2.20
Local-17	2.16	0.78	1801.28	233.27	6.50
Raj 4120	0.81	0.59	1091.13	104.91	0.96
Local-18	0.71	0.82	1033.55	140.71	0.69
PBW 34	0.92	0.98	1191.82	196.54	1.12
PDW 274	1.88	0.61	1664.92	166.24	5.18
PDW 291	0.72	1.60	1109.05	315.21	0.56
PDW 314	0.66	1.43	1042.32	262.29	0.49
DWL 5023	0.65	0.71	984.98	114.61	0.60
PDW 215	0.63	0.75	969.74	120.34	0.56
PDW 233	0.70	2.05	1147.24	437.34	0.43

OE, Optimum environment; HSE, Heat stress environment; STI, Stress tolerance index; SSI, Stress susceptibility index; MP, Mean productivity; Tol, Tolerance and MSTI, Modified stress tolerance index

Table S2. Stress indices of 34 genotypes under OE and HDSE conditions at Banda

Genotype	STI	SSI	MP	TOL	MSTI
Local-1	0.23	0.91	631.02	201.64	0.10
Local-2	0.29	0.91	719.44	231.19	0.17
Local-3	0.28	0.42	646.31	87.56	0.21
Local-4	0.87	0.48	1153.94	180.06	2.01
Local-5	1.06	0.62	1301.36	269.36	2.76
Local-6	0.56	1.03	1023.94	381.19	0.56
Local-7	0.48	0.53	862.80	149.96	0.59
Local-8	0.62	0.64	995.11	213.61	0.92
Local-9	0.42	0.84	848.44	247.31	0.37
Local-10	0.28	0.60	663.86	132.86	0.19
Local-11	0.22	0.43	579.19	80.44	0.13
HI-8759	1.09	0.66	1326.94	294.94	2.83
HI-8737	0.51	0.84	933.77	274.14	0.54
HI-8713	1.26	0.60	1410.14	285.14	3.86
HI-8777	1.04	0.58	1281.06	247.56	2.71
HI-8802	1.62	0.43	1567.48	220.86	7.16
HI-8627	1.03	0.58	1273.89	247.14	2.64
HI-8805	0.74	0.86	1132.89	339.39	1.14
Local-12	0.41	0.68	811.64	186.89	0.39
Local-13	1.03	0.54	1269.52	228.89	2.73
Local-14	0.68	0.53	1025.56	180.31	1.18
Local-15	0.13	1.08	505.69	198.94	0.03
Local-16	0.40	1.33	951.14	481.64	0.22
DBW 187	1.07	0.60	1302.69	262.81	2.82
Local-17	2.04	0.50	1771.11	293.61	10.81
Raj 4120	0.59	0.81	1003.27	280.64	0.75
Local-18	0.55	0.82	965.69	276.44	0.63
PBW 34	0.55	1.18	1058.06	464.06	0.47
PDW 274	1.64	0.55	1601.86	292.36	6.86
PDW 291	0.43	1.38	1000.44	532.44	0.23
PDW 314	0.43	1.24	951.19	444.56	0.27
DWL 5023	0.38	1.12	864.61	355.36	0.24
PDW 215	0.38	1.11	856.36	347.11	0.24
PDW 233	0.47	1.43	1069.48	592.86	0.26

OE, Optimum environment; HDSE, Combined heat – drought stress environment; STI, Stress tolerance index; SSI, Stress susceptibility index; MP, Mean productivity; Tol, Tolerance and MSTI, Modified stress tolerance index

Table S3. Stress indices of 34 genotypes under OE and HSE conditions at Jhansi

Genotype	STI	SSI	MP	TOL	MSTI
Local-1	0.16	0.51	383.50	70.00	0.08
Local-2	0.20	1.14	490.25	227.00	0.07
Local-3	0.18	0.63	414.25	95.50	0.09
Local-4	1.53	0.45	1165.12	186.77	7.12
Local-5	0.59	1.34	925.00	522.25	0.47
Local-6	1.06	0.67	1006.66	250.18	2.96
Local-7	0.24	1.22	558.25	281.50	0.09
Local-8	0.58	1.13	840.25	385.00	0.59
Local-9	0.31	0.97	585.25	223.00	0.20
Local-10	0.19	1.06	469.13	199.50	0.07
Local-11	0.28	1.11	572.00	255.50	0.13
HI-8759	0.54	0.74	727.13	201.75	0.73
HI-8737	0.43	0.85	668.50	219.25	0.43
HI-8713	0.51	1.13	787.25	360.50	0.45
HI-8777	1.69	0.49	1233.22	218.57	8.48
HI-8802	1.00	0.52	952.67	179.66	2.91
HI-8627	0.75	0.30	798.67	84.16	1.85
HI-8805	0.97	0.54	939.62	183.27	2.70
Local-12	0.64	0.61	770.00	173.00	1.10
Local-13	0.45	0.78	668.00	197.00	0.48
Local-14	0.33	1.18	641.50	308.50	0.18
Local-15	0.13	0.48	344.50	59.50	0.05
Local-16	0.29	1.10	589.00	262.00	0.15
DBW 187	1.77	0.54	1269.78	250.44	8.93
Local-17	0.90	0.55	907.16	179.18	2.32
Raj 4120	1.48	0.41	1138.66	166.18	6.79
Local-18	0.59	0.72	755.50	203.50	0.87
PBW 34	0.93	0.51	915.22	167.57	2.52
PDW 274	1.40	0.52	1124.50	209.50	5.69
PDW 291	0.45	1.30	787.25	429.50	0.28
PDW 314	0.48	0.86	703.25	231.50	0.52
DWL 5023	0.55	1.12	813.75	366.75	0.54
PDW 215	0.34	0.91	598.75	212.50	0.25
PDW 233	0.59	1.33	917.25	516.00	0.46

OE, Optimum environment; HSE, Heat stress environment; STI, Stress tolerance index; SSI, Stress susceptibility index; MP, Mean productivity; Tol, Tolerance and MSTI, Modified stress tolerance index

Table S4. Stress indices of 34 genotypes under OE and HDSE conditions at Jhansi

Genotype	STI	SSI	MP	TOL	MSTI
Local-1	0.12	0.71	354.24	128.51	0.06
Local-2	0.17	0.96	478.18	251.13	0.09
Local-3	0.15	0.70	392.57	138.87	0.09
Local-4	0.72	0.98	992.57	531.87	1.52
Local-5	0.47	1.13	897.01	578.24	0.48
Local-6	0.71	0.86	922.68	418.13	1.80
Local-7	0.21	1.00	547.93	302.13	0.13
Local-8	0.37	1.11	784.76	495.99	0.31
Local-9	0.23	0.96	552.07	289.37	0.16
Local-10	0.15	0.97	449.76	238.24	0.07
Local-11	0.21	1.02	546.18	307.13	0.12
HI-8759	0.25	1.10	631.43	393.13	0.14
HI-8737	0.27	0.98	613.49	329.26	0.22
HI-8713	0.40	1.02	755.49	424.01	0.44
HI-8777	1.08	0.80	1110.82	463.37	4.51
HI-8802	0.50	0.98	822.76	439.49	0.73
HI-8627	0.57	0.55	741.49	198.51	1.67
HI-8805	0.63	0.81	851.99	358.51	1.53
Local-12	0.47	0.75	718.24	276.51	0.91
Local-13	0.28	0.95	608.99	315.01	0.24
Local-14	0.26	1.04	617.82	355.87	0.18
Local-15	0.11	0.58	327.43	93.63	0.06
Local-16	0.25	0.95	572.74	294.51	0.19
DBW 187	1.12	0.83	1144.57	500.87	4.60
Local-17	0.49	0.93	796.26	400.99	0.76
Raj 4120	0.95	0.76	1021.99	399.51	3.65
Local-18	0.47	0.75	719.18	276.13	0.92
PBW 34	0.51	0.91	802.07	393.87	0.84
PDW 274	1.12	0.62	1064.38	329.74	6.03
PDW 291	0.35	1.11	762.93	478.13	0.29
PDW 314	0.41	0.79	679.82	278.37	0.66
DWL 5023	0.31	1.15	749.01	496.24	0.20
PDW 215	0.27	0.88	571.01	267.99	0.24
PDW 233	0.36	1.23	864.88	620.74	0.21

OE, Optimum environment; HDSE, Combined heat – drought stress environment; STI, Stress tolerance index; SSI, Stress susceptibility index; MP, Mean productivity; Tol, Tolerance and MSTI, Modified stress tolerance index
